# An Expert Fitness Diagnosis System Based on Elastic Cloud Computing

**DOI:** 10.1155/2014/981207

**Published:** 2014-03-02

**Authors:** Kevin C. Tseng, Chia-Chuan Wu

**Affiliations:** ^1^Product Design and Development Laboratory, Department of Industrial Design, College of Management, Chang Gung University, 259 Wenhua 1st Road, Guishan Shiang, Taoyuan 33302, Taiwan; ^2^Healthy Aging Research Center, Chang Gung University, Taiwan

## Abstract

This paper presents an expert diagnosis system based on cloud computing. It classifies a user's fitness level based on supervised machine learning techniques. This system is able to learn and make customized diagnoses according to the user's physiological data, such as age, gender, and body mass index (BMI). In addition, an elastic algorithm based on Poisson distribution is presented to allocate computation resources dynamically. It predicts the required resources in the future according to the exponential moving average of past observations. The experimental results show that Naïve Bayes is the best classifier with the highest accuracy (90.8%) and that the elastic algorithm is able to capture tightly the trend of requests generated from the Internet and thus assign corresponding computation resources to ensure the quality of service.

## 1. Introduction

The aging society is a continually growing and accelerating trend [1]. According to a world population report from the United Nations [2], there were approximately 810 million persons aged 60 years or over in the world in 2012, and this number will increase to more than 2 billion by 2050. Moreover, the United Nations estimates that 40% of older people live alone or only with a spouse. Among this older population, frailty, a status of multiple systems declining, producing negative outcomes (e.g., metabolic syndrome) [3–7], is a prevalent phenomenon. To help older people to counter this problem and have a healthier life, numerous studies have been proposed. In these studies, physical activity has been validated as one of the most effective and economical methods against aging [8–10]. It not only helps older people against metabolic syndrome and mood disturbance [11] but also enhances central nervous system health and cognitive functions [12]. It is obvious that physical activity has positive effects on health. However, moderate activity requires specialized knowledge from experts; without such knowledge, it will not enhance health status and may even cause injury. Now, along with the trend of the aging society, the fast-growing demands of physical activity can be expected, so expert resources to satisfy these needs are critical. Because expert resources are very limited and life is priceless [13], more cost-effective and flexible methods are required.

In recent years, cloud computing has been a hot topic. Cloud computing delivers reliable and high-quality service to users on a scalable and elastic infrastructure; it can be a set of heterogeneous computing units organized together, but it works like a homogenous single machine. Cloud computing systems can be easily scaled up and down, so it provides huge potential for solving complex problems and ensures quality of service (QoS). In supervised machine learning [14], a computing system automatically learns classification logics from data that are manually collected by experts, and when the learning is finished, the system runs intelligently, like an expert, and thus can be treated as an expert system. A traditional expert system is built on a single machine, and it is no longer able to handle the enormous number of requests from the Internet. However, when an expert system is combined with cloud computing, it can deal with the massive demands from the Internet. In this paper, we aim to provide an expert system on a cloud infrastructure. This system provides fitness status diagnoses based on a supervised machine learning technique, and it can also automatically scale up and down based on a resource allocation algorithm for improving the QoS. The remainder of this paper is organised as follows: Section 2 presents related work, Section 3 describes the proposed system, Section 4 shows the experimental results, and Section 5 draws conclusions.

## 2. Related Work

Physical activity is a critical factor in healthcare, and it has direct relevance for mortality. According to a report from the World Health Organization (WHO) in 2008, insufficient physical activity is among the top four risk factors for mortality. About 3.2 million deaths are caused by insufficient physical activity, so it is recommended that people participate in 150 minutes of moderate physical activity a week. Moreover, in regions with the most insufficient physical activity, such as the Americas and the Eastern Mediterranean region, almost 50% of women are insufficiently active, while the prevalence for men is 40% [15]. However, a more detailed survey conducted by Kokkinos in 2012 [16] indicates that, although the threshold for a significant reduction in mortality risk appears to be at a caloric expenditure of approximately 1,000 Kcal per week, this activity must be customised to meet individual needs. It also indicates that the risk of death during physical activity for a person with a sedentary lifestyle is 2.5 to 30 times higher than that of a person with habits of regular physical activity. Given this supportive evidence, we can see the urgency of providing customised and intelligent healthcare services for users to monitor their physical status and thus to enhance it for a better life.

### 2.1. Current Expert Systems in Healthcare

Expert systems in the medical domain have been continually developed since 1970. De Dombal et al. proposed a computer-aided system, which performs diagnosis of acute abdominal pain [17]. In 1970, the computation capacity of computers was quite limited, so the nature of the model in expert systems was naïve [18], but today, with the huge advance in technology, more sophisticated and advanced approaches are proposed.

Seto et al. [19] presented a phone-based expert system for telemonitoring in 2012; this system detects heart failure according to a set of predefined rules that are constructed and iteratively modified by heart failure clinicians. The process of defining rules is time consuming and not computationally flexible. However, this system can be distributed by smart phone, and it can be widespread in a client-server mode; when a rule set on the server side is modified, every client can get the latest version through the Internet, so it still maintains some efficiency. Ongenae et al. proposed another rule-based expert system [20], but in contrast to the research of Seto et al., the rule set of the system is automatically generated from the patient's physiological data through a decision tree algorithm [21]. They proposed a system framework and implemented a specific use case, using a decision tree to detect systemic inflammatory response syndrome (SIRS). In this system, clinicians no longer need to define static rules nor modify them; they only need to identify whether the patient has SIRS or not. Next, the physiological data of the patient can be collected through an electronic sensor, and a decision tree [22] can be applied to the physiological data for rule learning. This system is more flexible and more cost effective, which reduces the intervention of clinical experts. In the research of Lo et al. [23], more complex methods were used for daily diet recommendations. They used micro sensors combined with RFID (radio-frequency identification) to collect the daily vital data of a user, and these vital data are examined by an expert and corresponding diet suggestions are recommended for health management. Then, several techniques, TF-IDF (term frequency-inverse document frequency) [24], K nearest neighbor (KNN) [25], latent semantic analysis (LSA) [26], medical ontology [23], and the curative food stemming mechanism [23], are applied for supervised learning. It is obvious that the trend of the system design is moving toward self-learning based upon data that are systematically collected with little intervention of experts. However, we rarely see a self-learning system proposed on the topic of physical fitness. Although Acikkar et al. [27] proposed an expert system based on support vector machine (SVM) [28] for detecting the fitness level of the athlete; the data they used to train the SVM [28] are inconvenient to measure for ordinary people (e.g., the speed of the user and the grade of the user). Furthermore, to the best of our knowledge, an expert system built on a cloud computing scale is also rare. Current expert systems in healthcare are still built on a single machine in a client-server mode [29]. However, with the continually growing healthcare requests, we believe that this mode cannot cover the future demands and challenges.

### 2.2. Motivation

The motivation of this work comes from the urgent need for physical fitness diagnoses for improving the health status of older people, and current state-of-the-art expert systems do not address this issue. Moreover, to help older people diagnose their health status, we cannot rely on conventional method that applies a universal standard for measuring fitness level [30]. The diagnosis must be customised according to the elder person's personal features, such as gender, age, and fitness level. In the research of Belza et al. [31], personal instructors were involved; they can give more appropriate adjustments during fitness measurement. However, this approach is expensive and cannot be deployed in a cost-effective way (e.g., without the intervention of an expert). This led us to build an automatic expert fitness diagnosis system that has not been proposed before. This system makes customised diagnosed methods for elders, and it is deployed on the cloud computing scale. It can automatically allocate computational resources to maximise the QoS (quality of service) to the client while saving computation resources on the server side.

## 3. Proposed System

### 3.1. Overview

**Figure 1:**
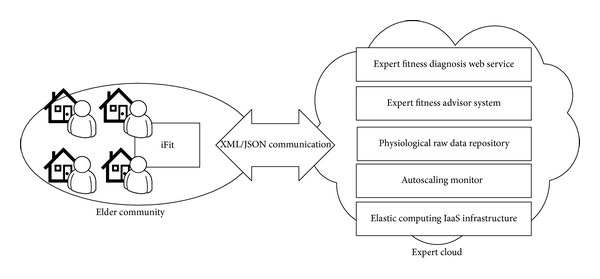
Overview of expert cloud architecture.

Figure 1 illustrates the overview of the proposed cloud architecture. The iFit [32] is a user-friendly platform for fitness promotion in the elder community. It measures the user's degree of fitness through a game-like activity. The expert cloud is a prototype cloud system that provides expert fitness diagnoses through a Web service. This Web service receives physiological data and returns the corresponding fitness level. The Web service is deployed on an elastic cloud computing IaaS (infrastructure as a service) infrastructure, and the autoscaling mechanism dynamically supplies computing resources according to external requests from the Internet. The expert fitness diagnosis system is the core of the expert cloud, and it makes inferences based on a knowledge database. This system is built by machine learning techniques, and the analytical results of user fitness level are learned from a physiological raw data repository. The repository stores the general physiological data of users without identity information. iFit communicates with the expert cloud via an XML/JSON form. When users measure their fitness on iFit, it sends the users' physiological data to the expert cloud, the expert cloud returns the corresponding fitness level to iFit, and then iFit gives fitness suggestions to the user.

### 3.2. iFit

**Figure 2:**
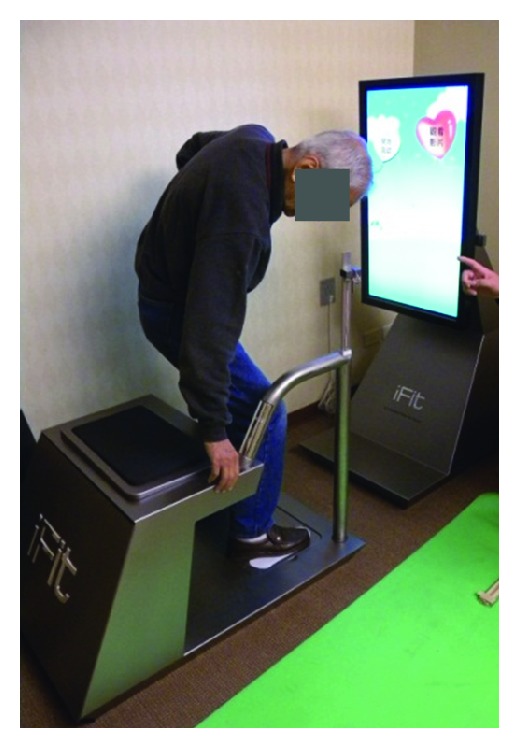
The iFit for physical fitness measurement and promotion.

**Figure 3:**
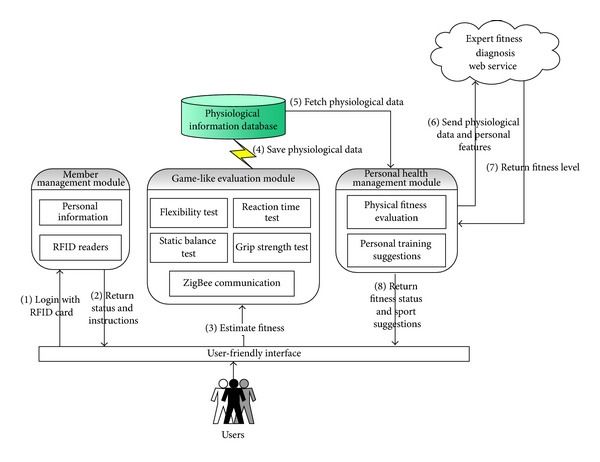
The system architecture of iFit.

iFit [32] is designed as a platform for elders to monitor and enhance their physical fitness (see Figure 2). It includes a monitor, two bases, a cushion, a handrail, and a stand pad. The system architecture of iFit contains three modules, and their relationships are illustrated in Figure 3. When an elder is using iFit, the elder will first use an RFID card that represents the elder's identity to log in to the system, and then the member management module will search for the corresponding identify information and display it on the screen. This module will also provide instructions to guide the user during the process. Next, a game-like evaluation module provides four game-playing scenarios, stealing eggs, making dumplings, jet skiing, and parachuting, which test flexibility, grip strength, balance, and reaction time, respectively. The four games are designed to motivate elders to engage in the test. When the tests are finished, the results will be saved into a physiological database. Finally, a personal health management module will fetch the physiological data and combine them with the elder's personal features, sex, age, and BMI. These data will be sent to the expert fitness diagnosis Web service for a fitness diagnosis, and the analysed fitness level of the elder will be sent back and displayed on the screen. The fitness levels are classified as strong, moderate, and weak. They are used to represent the level of health of the elder's flexibility, grip strength, balance, and reaction time. According to the fitness levels of each test, corresponding sport suggestions will be also displayed on the screen.

The difference between the iFit in this work and previous work [32] is that the previous work classified the fitness level of an elder according to a comparison with the performance of another group of elders of the same age. However, this scale is too general, so it fails to capture differences in age, BMI, and sex. To design a universal standard accounting for these features, voluminous efforts are required. Thus, an expert system based on machine learning is provided to solve this problem; the personal features of elders will be incorporated during fitness level classification so more accurate fitness levels can be achieved.

### 3.3. Expert Fitness Diagnosis System Construction

**Figure 4:**
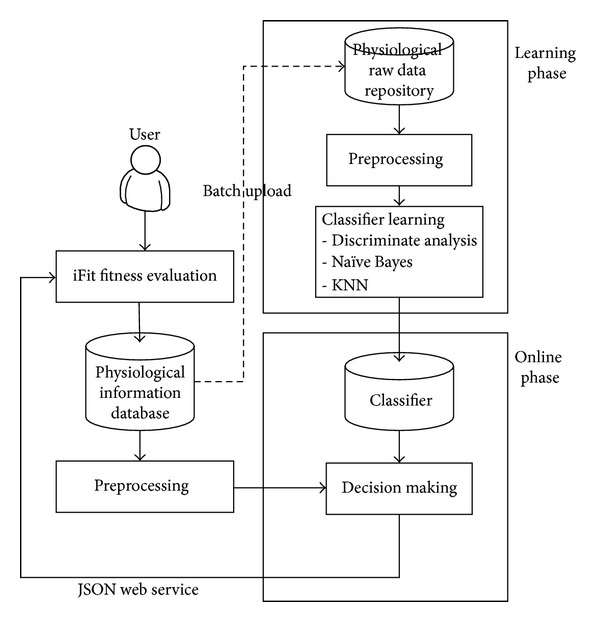
Process of classifier learning and decision making.

The process of building the expert fitness diagnosis system is illustrated in Figure 4. Expert fitness diagnosis is an automatic classification system. It includes a set of classifiers. In the learning phase, iFit extracts a user's raw fitness data and stores them in the physiological information database. These data will be uploaded to the physiological raw data repository on the cloud before classifier learning. We investigate three classification techniques, KNN [33], Naïve Bayes [18], and discriminate analysis [34]. In preprocessing, the data will be normalised into a form that the classification techniques can process. Then, after classifier learning is finished, a classifier will be built and stored. In the online phase, iFit extracts the physiological data from the user and sends it to the classifier through JSON communication. The classifier will classify the fitness level of the user and return the result to the user.

#### 3.3.1. Data Preprocessing and Classifier Learning

The preprocessing task normalises the raw data of the user into an instance that consists of a set of features; these features are described in Table 1. For a 65-year-old female with a weight of 50 kgs and a BMI value of 25, when she is using iFit, her flexibility, reaction time, grip strength, and balance will be tested and stored. Then the preprocessing will normalise the data into a sequence of feature values as follows: instance_*i*_ = [0, 25, 50, 25, 29.4, 501, 12.2, 5.86]. The last four values tested by iFit are not intuitively interpretable, but these data can be automatically processed by classifier learning algorithms.

Next, when preprocessing is completed, the raw data will be converted into instances. Matlab is used to analyse the performance of KNN, Naïve Bayes, and discriminate analysis. However, since Matlab cannot be fully integrated with Java, Weka [22], which is fully written in Java, is applied to build the classifiers. In Weka, all instances share the same data format, attribute-relation file format (ARFF), and various algorithms can be applied to them to train a classifier. When the training is over, the classifier is stored and deployed on a Tomcat Web application server. A JSP (JavaServer Pages) Web page is built as an interface to receive a JSON (JavaScript object notation) request posted by iFit and return the classification result.

### 3.4. Autoscaling Mechanism

In a cloud computing system, computational resources can be dynamically allocated. However, to determine how many computational resources are needed, a detailed analysis is necessary. If the allocated resources are greater than required, it wastes electricity and produces unnecessary carbon dioxide. If the allocated resources are less than required, it degrades the quality of service. Hence, this section presents an algorithm based on the Poisson distribution that predicts the required computational capacity according to the number of past requests from the Web service.

Let a period of time be divided into *n* intervals and let *t*_*i*_ represent an arbitrary time interval. In *t*_*i*_, there are several classification requests from the Internet, and their number is denoted by *Q*_*i*_. In a cloud computing system, there are a number of running computational units in *t*_*i*_; these units are denoted by *U*. We assume that each unit has an equal computational capacity that can process *C* requests, so the total computation capacity in *t*_*i*_ is *U* × *C*, where *U* × *C* ≥ *Q*_*i*_. In *t*_*i*_, the required computational capacity in *t*_*i*+1_ is predicted based on the Poisson distribution. The probability density function of the Poisson distribution is expressed as follows:

(1) $$\begin{array}{c} P\left(X=k\right)=\frac{{\lambda_{i}}^{k}e^{-\lambda_{i}}}{k!}, \end{array}$$

where *X* represents the number of requests and *λ*_*i*_ represents the average number of requests calculated at *t*_*i*_. Equation (1) calculates the probability of *k* requests generated from the Internet. To calculate *λ* more precisely, we apply an exponential moving average, described as follows:

(2) $$\begin{array}{c} \lambda_{i}=\alpha \times Q_{i}+\left(1-\alpha \right)\times \lambda_{i-1}, \end{array}$$

(3) $$\begin{array}{c} \alpha =\frac{2}{w+1}, \end{array}$$

where *α* ranges from 0 to 1. Equation (2) calculates *λ*_*i*_ according to *Q*_*i*_ and the previous *λ*_*i*−1_, and *α* is used to control the relative weights of *Q*_*i*_ and *λ*_*i*−1_. A higher *α* value indicates a lower contribution from *λ*_*i*−1_ and a higher contribution from *Q*_*i*_. *α* is calculated by (3) and *w* denotes time period; a higher value of *w* indicates a lower value of *α*. To predict the required computation capacity at *t*_*i*+1_, we need to define some parameters: (a) *I* denotes the maximum number of computational units which can be increased or decreased from the current running units *U*; (b) *Thre*_*i*_ and *Thre*_*d*_ range from 0 to 1, and they represent the threshold for increasing and decreasing computational units.

In Algorithm 1, Step 1 calculates *λ*_*i*_ given the requested data in the past *Q*_*i*_. Steps 2 to 10 are an iterative process. In each iteration, the probability of the need to increase *i* units and decrease *i* units will be estimated. If the probability of increasing the units is higher than the threshold *Thre*_*i*_, the computational units will be increased. If the probability of decreasing the units is higher than the threshold *Thre*_*d*_, the computational units will be decreased. If both probabilities are lower than their thresholds, the running units *U* are unchanged.

## 4. Experimental Results

### 4.1. Data Generation

To collect the physiological data from users, we recruited 85 adults aged 55 to 85 years old, 36 males and 49 females, from the Chang Gung Health and Culture Village in Taiwan. First, the elders were asked to log in to iFit and use it to test their grip strength, balance, flexibility, and reaction time. Second, a professional trainer classified the result of each test into the strong, moderate, and weak categories according to his knowledge. Finally, the fitness level given by the trainer and the physiological data collected from iFit are combined and treated as training data for classifier evaluation.

### 4.2. Experimental Setup

The expert fitness diagnosis system is built upon a cloud platform that consists of five server workstations. The five workstations share the same hardware standard: CPU E31270 V2, 3.5 GHz, 8 GB memory, and the Windows Server 2003 operating system. One of the workstations serves as a master node with an autoscaling mechanism, and the other workstations are treated as slave nodes. The expert fitness diagnosis Web service is deployed on a Tomcat Web server, and all workstations hold the same Web service.

### 4.3. Performance Metrics

Precision, recalled from *F*1 [35], is used to evaluate the classifier in this paper. The measures are shown in Table 2, with the definitions shown below:

 
*C*: the total number of categories,
 
*c*: a category,
 TP_*c*_: the number of users correctly classified while the fitness level is *c*,
 FP_*c*_: the number of users incorrectly classified while the fitness level is *c*,
 FN_*c*_: the number of users belonging to fitness level *c* but incorrectly classified.

The mean absolute percentage error (MAPE) [36] is used to evaluate the performance of the elastic allocation algorithm. The equation is described as follows:

(4) $$\begin{array}{c} \frac{1}{n}\overset{n}{\underset{t=1}{\sum }}\left|\frac{A_{t}-F_{t}}{A_{t}}\right|*100, \end{array}$$

where *A*_*t*_ represents the actual value, *F*_*t*_ denotes the predicted value, and *n* is the total number of observations. If the *elastic allocation algorithm* works well, the MAPE will be very close to zero; otherwise, the value of MAPE will be very large.

### 4.4. Classifier Performance

The performance of classifiers is tested by K-fold cross-validation, and the average *F*1 of each trial is selected to represent the total performance. The results are presented in Table 3.

Naïve Bayes has the best accuracy among other methods. The performance of discriminate analysis is lower than that of Naïve Bayes but much higher than that of KNN. KNN has the worst performance. This shows that, when trying to classify the fitness level of an elder, it cannot make decisions according to records of similar individuals. According to the experimental results, the expert fitness diagnosis system should include four Naïve Bayes classifiers. However, if a more accurate technique is found, say SVM, it can be combined with Naïve Bayes or replace it if better performance can be obtained.

### 4.5. Autoscaling Mechanism Performance

**Figure 5:**
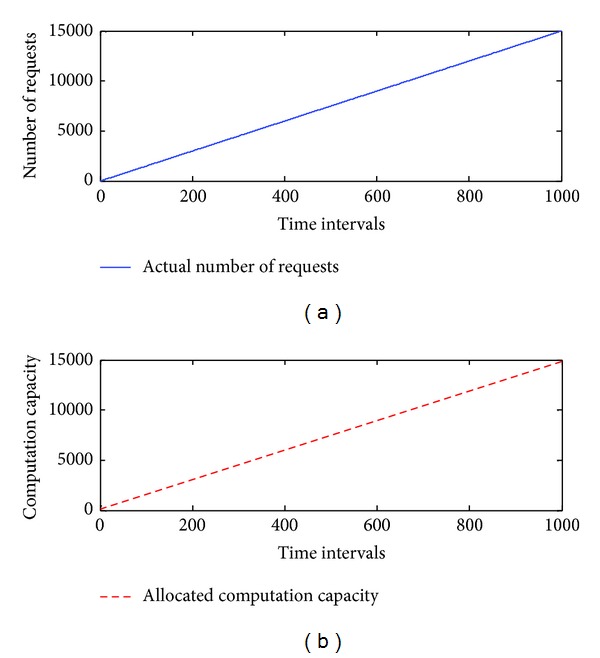
The simulation results of a linear pattern.

**Figure 6:**
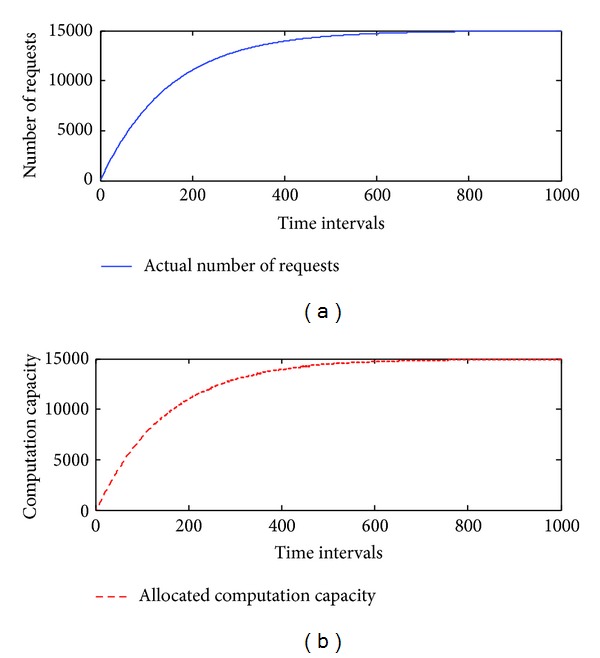
The simulation results of a logarithmic growth pattern.

**Figure 7:**
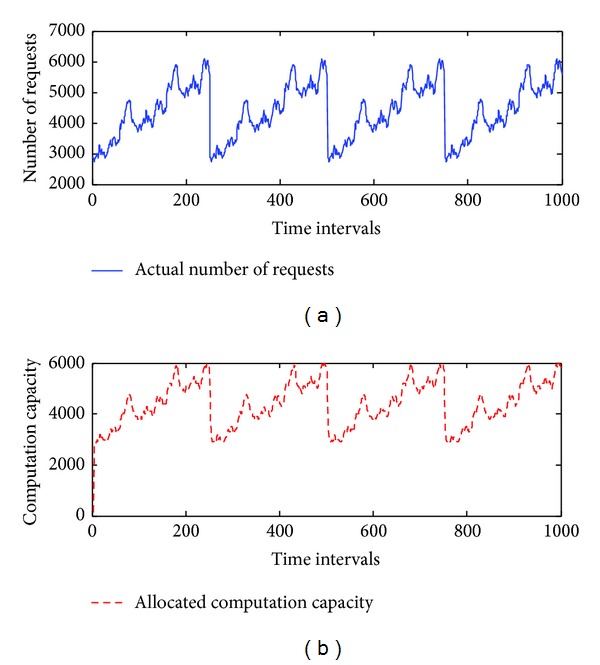
The simulation results of a repetitive pattern.

**Figure 8:**
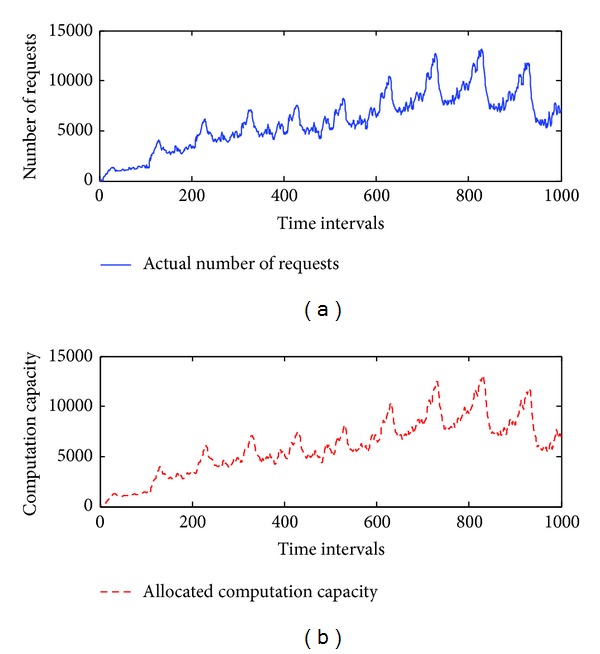
The simulation results of a combined pattern.

To estimate the performance of *elastic allocation algorithm*, we start from a simulation approach. The patterns include linear, logarithmic growth, repetitive, and combined (linear and repetitive) patterns. They are used to simulate the number of requests from the Internet. The maximum number of requests is set at 15,000, the minimum number of requests is set at 0, and *C* is set to 100. We assume that there are 150 machines running on the cloud infrastructure. The initial value of *U* is set at 1. *Thre*_*i*_ and *Thre*_*d*_ are set at 0.2. The total number of time intervals is set at 1,000. The simulated results are presented in Figures 5, 6, 7, and 8, and the performance is described in Table 4.

The simulation results demonstrate that the *elastic allocation algorithm* is able to capture the trend of actual requests from the Internet and provide accurate computation capacity. In Table 4, 2.68% additional computation capacity is provided in the linear pattern and 1.1% for the logarithmic pattern. The combined pattern is the hardest pattern to predict; around 10% additional computation capacity is provided.

Next, we focus on a more realistic experiment. The repetitive pattern is used and simulated on our cloud platform, which consists of five computers. To save time, we selected time intervals ranging from 75 to 100 in Figure 7 for the experiment. *C* is set to 1,000, so each computer is set to handle 1,000 requests. We wanted to test whether the *elastic allocation algorithm* could effectively allocate the five computers. First, the total running time for processing all the requests was estimated. Then we assigned a different number of computers to process the requests simultaneously and to test whether the total running time could be reduced. The results are presented in Table 5.

In Table 5, it is obvious that the more computers we assigned, the faster running time could be obtained. When five computers are always assigned for processing requests, the total running time is the shortest. However, the loading is not always large enough to utilise five computers on the cloud. The computation capacity should be allocated when it is necessary. The *elastic allocation algorithm* is applied here to run the same task, and the results are presented in Table 6.

Table 6 shows that *elastic allocation algorithm* can intelligently allocate the computers; the average number of allocated computers is 2.9, but the running time is close to the running time of five computers working simultaneously.

## 5. Conclusion

This paper presents a cloud-based expert fitness diagnosis system; it measures a user's fitness level based on iFit with a combination of machine learning techniques. Discriminate analysis, Naïve Bayes, and KNN are utilised to build this system. This system classifies the user's fitness level into strong, moderate, and weak, and then iFit can give corresponding sport suggestions to the user. To improve the processing time of requests, this work presents an *elastic allocation algorithm* to allocate computation resources automatically. The experimental results show that Naïve Bayes has the highest classification accuracy and that the *elastic allocation algorithm* is able to capture the trend of requests in several patterns. It dynamically increases computation capacity when the loading is high and decreases it when the loading is low. Thus, this system is elastic enough to cover numerous requests from the Internet while providing high precision and a customised fitness diagnosis.

## Figures and Tables

**Algorithm 1:**
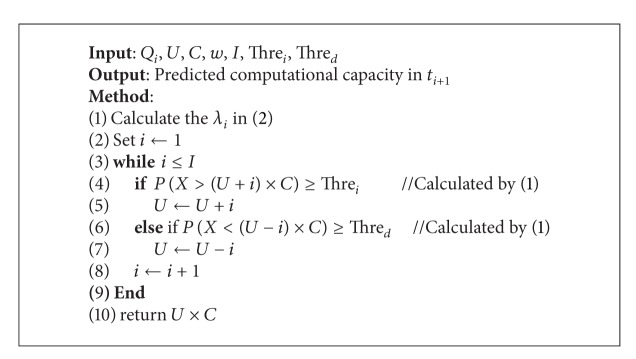
Elastic allocation.

**Table 1:** Descriptions of features.

Feature	Description
Gender	The gender of the user (male: 1, female: 0)
Age	The age of the user (years)
Weight	The weight of the user (kg)
Body mass index	The BMI of the user
Flexibility	The flexibility of the user tested by iFit
Reaction time	The reaction time of the user tested by iFit
Grip strength	The grip power of the user tested by iFit
Balance	The balance of the user tested by iFit

**Table 2:** Evaluation measures.

Measure	Description
Precision	$\frac{{\text{TP}}_{c}}{{\text{TP}}_{c}+{\text{FP}}_{c}}$
Recall	$\frac{{\text{TP}}_{c}}{{\text{FP}}_{c}+{\text{FN}}_{c}}$
*F*1	$\frac{2\times \text{Precision}\times \text{Recall}}{\text{Precision}+\text{Recall}}$

**Table 3:** Classification performance (average *F*1 of 4-fold cross-validation).

	Flexibility	Balance	Grip strength	Reaction time
KNN	0.70	0.88	0.82	0.34
Naïve Bayes	0.87	0.95	0.97	0.84
Discriminate analysis	0.82	0.84	0.86	0.71

**Table 4:** The MAPE values of simulation results.

Pattern	MAPE
Linear	2.68%
Logarithmic growth	1.10%
Repetitive	3.73%
Combined	9.19%

**Table 5:** The performance of multiple computers running simultaneously.

Number of assigned computers	Total running time	Average number of assigned computers in each time interval
1	743,870 ms	1
2	443,644 ms	2
3	421,508 ms	3
4	362,589 ms	4
5	316,873 ms	5

**Table 6:** The performance of the *elastic allocation algorithm*.

Elastic allocation mechanism	Total running time	Average number of assigned computers in each time interval
*Elastic allocation algorithm *	328,885 ms	2.9
